# Visible Thrombolysis Acceleration of a Nanomachine Powered by Light-Driving F_0_F_1_-ATPase Motor

**DOI:** 10.1186/s11671-015-0918-z

**Published:** 2015-05-21

**Authors:** Xiaoxia Duan, Lifeng Liu, Weijian Jiang, Jiachang Yue

**Affiliations:** The National Laboratory of Biomacromolecules, Institute of Biophysics, Chinese Academy of Sciences, Beijing, 100101 China; Department of Interventional Neuroradiology, Beijing Tiantan Hospital, Capital Medical University, 6 Tiantan Xili, Beijing, 100050 China; Department of Neurology, Liaocheng People’s Hospital and Liaocheng Clinical School of Taishan Medical University, Liaocheng, Shandong 252000 China; New Era Stroke Care and Research Institute of The Second Artillery General Hospital PLA, 16 Xinjiekouwai Avenue, Beijing, 100088 China

**Keywords:** F_0_F_1_-ATPase, Nanomachine, Urokinase, Thrombolysis

## Abstract

We report on thrombolysis acceleration of a nanomachine powered by light-driving δ-subunit-free F_0_F_1_-ATPase motor. It is composed of a mechanical device, locating device, energy storage device, and propeller. The rotory δ-subunit-free F_0_F_1_-ATPase motor acts as a mechanical device, which was obtained by reconstructing an original chromatophore extracted from *Rhodospirillum rubrum*. We found that the bioactivity of the F_0_F_1_-ATPase motor improved greatly after reconstruction. The zeta potential of the nanomachine is about −23.4 mV. Cytotoxicity induced by the nanomachine was measured using cell counting kit (CCK)-8 assay. The A549 cells incubated with different fractional concentrations of the nanomachine within 48 h did not show obvious cytotoxicity. The locating device helps the nanomachine bind to the thrombi. Energy was easily stored by exposing the nanomachine to 600-nm-wavelength irradiation, which promoted activity of the motor. The rotation of the long propeller accelerated thrombolysis of a blood clot in vitro in the presence of urokinase (UK). This result was based on visual inspection and confirmed by a series of tests.

**Electronic supplementary material:**

The online version of this article (doi:10.1186/s11671-015-0918-z) contains supplementary material, which is available to authorized users.

## Background

Stroke remains a dangerous disease throughout the world and more than 5.7 million people experience an ischemic stroke each year [1]. Ischemic stroke is also the most common cause of permanent disability [2]. Due to an aging population, the rate of ischemic stroke is increasing each year [3]. Evidence-based medical therapies of acute ischemic stroke mainly include intravenous tissue-type plasminogen activator (tPA), intra-arterial thrombolysis, or interventional techniques [4, 5]. Although the American Heart Association/American Stroke Association (AHA/ASA) stroke guidelines were recently updated to support the use of tPA in selecting patients up to 4.5 h after symptom onset [6], many patients cannot get to a hospital within this time window. In addition, administration of systemic therapy increases the risk of bleeding [7–9]. Intra-arterial thrombolysis has extended this time window to 6 h after symptom onset [10], but for many medical institutions, the development and popularization of this complex multilateral cooperation technology is still restricted because of poor patient awareness, intention, and other reasons [11–13]. Blocked blood vessels can be opened using interventional techniques, but it requires experienced operators for successful implementation. Furthermore, individual risk factors are unavoidable, which makes catheter intervention an infeasible approach.

Reopening occlusive blood vessels safely and efficiently remains a very challenging work in patients with acute ischemic stroke or coronary heart disease. Our study focused on nanotechnology-assisted thrombolysis to explore a new direction in the field of thrombotic diseases research, such as developing new drugs. Compared with traditional thrombolytic methods, the nanotechnology devices are advantageous; they combine the effects of biochemical enzymolysis of a pure drug with the mechanical force of a nanodevice. Active research is currently working on the development and construction of nanobiomedical devices, which may overcome the shortcomings of traditional therapy.

F_0_F_1_-ATPase is a type of rotating molecular motor that is widely present in nature and has been discussed as a micro-mixer in the past [14]. In this study, a δ-subunit-free F_0_F_1_-ATPase motor was generated by reconstructing the original chromatophore extracted from *Rhodospirillum rubrum* as previously described [15]. The propeller and thrombus-locating device were introduced by biochemical reaction, and the nanomachine was subsequently constructed successfully. Rotation of the propeller can accelerate thrombolysis in a urokinase environment. Moreover, this nanomachine can work continuously due to stored energy that can be replenished when the device is exposed to light. In this study, the device induced an efficient and accelerated thrombolytic process.

## Methods

### Chemical and Materials

The antibody against the β-subunit was prepared according to a previous study [16] and purified according to the study by Su et al. [17]. n-biotin, ATP, LDH, NADH, and fibrinogen (F8630) were purchased from Sigma (Sigma-Aldrich, St. Louis, MO, USA) as well as a mouse monoclonal anti-fibrinogen antibody (Sigma-Aldrich, St. Louis, MO, USA). The protein nanoparticle was provided by Wuhan Institute of Virology, Chinese Academy of Sciences. Lipid-biotin was purchased from Avanti (Avanti Products, Doral, FL, USA). Fluorescein isothiocyanate (FITC) was purchased from Molecular Probes (Eugene, OR, USA). Human whole blood was drawn from healthy volunteers by sterile venipuncture. Informed written consent was obtained from all participants, and the protocol of the study was approved by the Ethical Committee of the Capital Medical University.

### Preparation of Blood Clots

Briefly, 2 ml of human peripheral blood was drawn from healthy volunteers by sterile venipuncture and was injected rapidly into a polypropylene tube (Shuanghe pharmaceutical company, Beijing, China) with a length of 20 cm and internal diameter 3 mm. This type of tube is biocompatible and initiates coagulation easily. Both ends of the tube were immediately clipped tightly and incubated at 37 °C overnight in order to achieve maximal clot retraction and stability [18].

After the incubation, long cylindrical clots along the inner diameter of approximately 2 mm were obtained once they were pushed out of the tubes. The long clots were cut into small pieces with a 2 mm width and diameter at the time of the experiment.

### Construction of Nanomachine

The nanomachine is composed of three parts: an energy storage device, power device with a long propeller, and locating device. The construction of this nanomachine was initiated by removing the F_1_ from chromatophore.

### Reconstruction of F_0_F_1_-ATPase Molecular Motor

The chromatophore containing F_0_F_1_-ATPase was prepared as previously described [19]. It was incubated with 2 M LiCl, 0.1 mM Tricine-NaOH, 10 mM MgCl_2_, and 1 mM ATP for 20 min at 4 °C and then washed twice with TSM buffer by centrifugation. This step removed most of the F_1_ and clearly isolated the δ-subunit from F_0_.

**Fig. 1:**
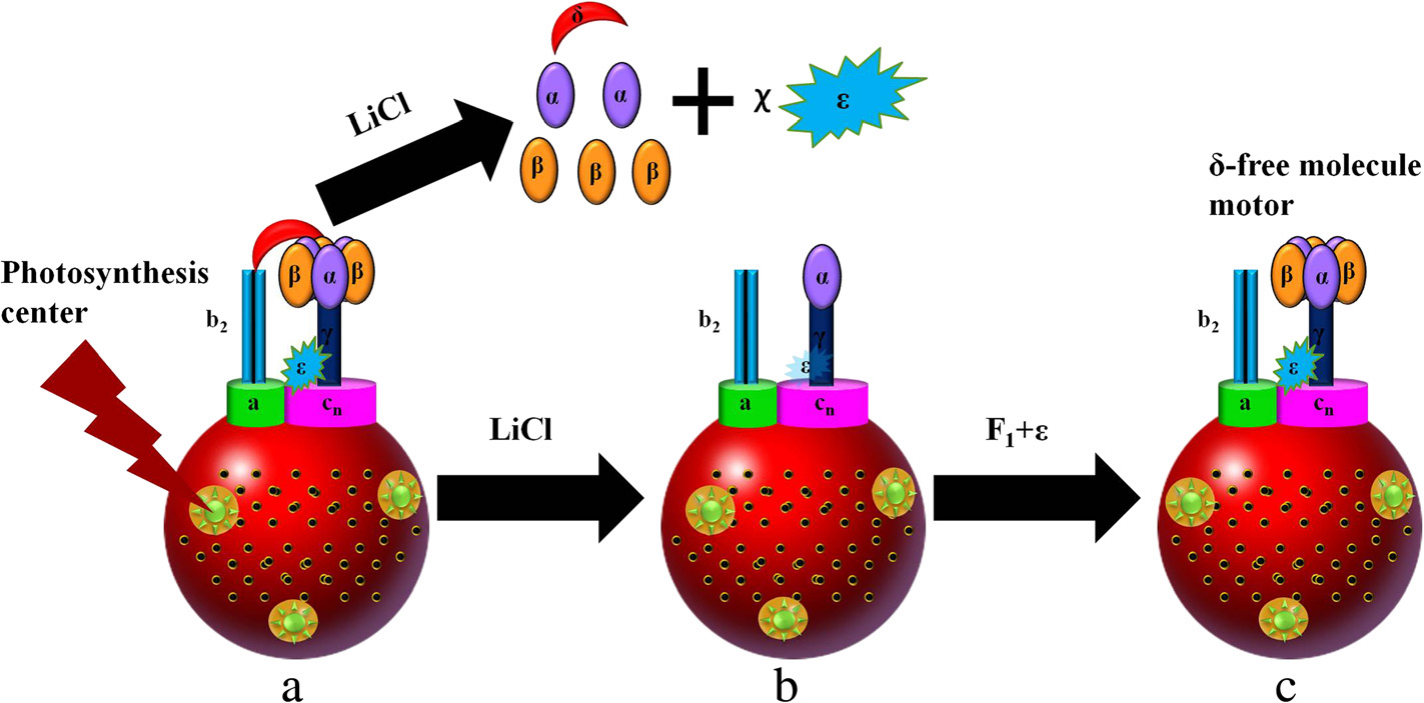
Reconstruction procedures for the δ-subunit-free F_0_F_1_-ATPase molecular motor. **a** The original holoenzyme ATPase molecular motor. **b** Subunit-free chromatophore. **c** The δ-free F_0_F_1_-ATPase molecular motor. The δ-subunit and most of the other subunits were removed from the original holoenzyme ATPase molecular motor (chromatophore) in LiCl solution and B was attained. The alternative PS3 F_1_ (α_3_β_3_γ) was added into the reconstruction system and ε-subunit was complemented at the same time, thus generating the δ-free F_0_F_1_-ATPase molecular motor

Reconstruction was initiated by removing F_1_ from chromatophore followed by grafting the alternative PS3 F_1_ (α_3_β_3_γ) onto it (Fig. 1). At the same time, ε-subunit was complemented into the reconstruction system. The PS3 F_1_ (α_3_β_3_γ) and the ε-subunit were prepared based on previously described methods [17, 20–22]. Next, 5 μg of chromatophores lacking F_1_, 10.4 μg ε-subunit, and 50 μg purified F_1_ (α_3_β_3_γ) were incubated at 25 °C for 12 h in reconstitution buffer containing 50 mM Tricine-NaOH (pH 8.0), 4 mM ATP, 25 mM MgCl_2_, 1 mM dithiothreitol, and 10 % glycerol. The retained system was centrifuged at 180,000 *g* for 30 min. The precipitate was resuspended in buffer (containing 50 mM Tricine-NaOH, pH 8.0, 10 % glycerol, and 25 mM MgCl_2_) and washed three times with the same buffer followed by centrifugation after each step. An artificial δ-subunit-free F_0_F_1_-ATPase within chromatophore was successfully reconstructed. A schematic diagram of the reconstruction process is shown in Fig. 1.

### Installing Propeller for Nanomachine

The propeller of the molecular motor was an anti-β-subunit polyclonal antibody-n-biotin-avidin-n-biotin-protein nanoparticle. To construct this propeller, 44.2 μl of 17 μM anti-β-subunit antibody and 3.8 μl of 1 mM n-biotin were incubated for 1 h, which formed the antibody-n-biotin complex. Then, 147 μl of 29 μM avidin was added and incubated at room temperature for 5 min, followed by incubation with 8.5 μl of 350 μg/ml protein nanoparticle and 1.5 μl of 1 mM n-biotin at room temperature for 1 h. Next, 48 μl [(polyclonal antibody against the β subunit)-n-biotin-avidin] and 10 μl n-biotin-protein nanoparticle were added and incubated at room temperature for 5 min. These steps resulted in the connection of [(polyclonal antibody for the β subunit)-n-biotin-avidin-n-biotin-protein nanoparticle] through long, whole links to the molecular motor. The final nanomachine can produce a stronger stirring force compared with the molecular motor alone due to the long propeller.

### Installation of the Locating Device

Briefly, 400 μl of 0.5 μg/μl chromatophores and 3.2 μl of 5.5 mM lipid-biotin were incubated at 4 °C for 25 min. Then, 632 μl of 29 μM avidin was added and incubated at room temperature for 5 min, which formed the chromatophore-lipid-biotin-avidin complex. Subsequently, 210.1 μl of 17 μM anti-fibrinogen antibody and 17.9 μl of 1 mM n-biotin were incubated at room temperature for 1 h, n-biotin-anti-fibrinogen antibody complex formed. Finally, 1035.2 μl of the chromatophore-lipid-biotin-avidin complex and 228 μl of the n-biotin-anti-fibrinogen antibody were mixed and incubated at room temperature for 5 min. The anti-fibrinogen antibody easily reacts with the fibrinogen in the thrombi and works as a locating device.

### Assembling Nanomachine

For assembly, 26 μl of anti-β subunit polyclonal antibody-n-biotin-avidin-n-biotin-protein nanoparticle complex was mixed with 1263.2 μl of molecular motor-lipid-biotin-avidin-n-biotin-anti-fibrinogen antibody complex and incubated at room temperature for 5 min followed by centrifugation at 35,000 rpm (180,000 *g*) for 30 min. The precipitate was finally resuspended in light-irradiation buffer [10 mM Tricine-NaOH (pH = 8), 2 mM MgCl_2_, and 50 mM KCl].

### Measurement of Bioactivity of the Reconstructed δ-Free F_0_F_1_-ATPase

The hydrolysis activity of the holoenzyme and the reconstructed molecular motor was measured according to previously described methods [17, 23]. In general, the efficiency of the reconstructed molecular motor was determined by the percentage of ATP hydrolysis. The rate of ATP hydrolysis was measured by the disappearance of NADH. Measuring the fluorescence intensity of NADH at 340 nm can indirectly reflect the activity of a hydrolytic enzyme because the maximum absorption peak of NADH is 340 nm. Fluorescence intensity was measured at 37 °C using a UV-2101PC Shimadzu Spectrophotometer (Shimadzu Corp., Tokyo, Japan).

### The Size Distribution and Zeta Potential

The size distribution and zeta potential were examined by Zetasizer Nano-ZS (Malvern Instruments Ltd., Worcestershire, UK). The analysis software was Zetasizer software version 7.02 supplied by the manufacturer (Malvern Instruments Ltd., Worcestershire, UK). The samples were diluted 30-fold in water. Dynamic light scattering (DLS) was measured at 25 °C using a helium–neon laser at the wavelength of 633 nm, detection angle of 173°.

### Cytotoxicity of the Nanomachine

The cytotoxicity of the nanomachine was assessed by cell counting kit (CCK)-8 assay (Beyotime, Beijing, China). The A549 cells were seeded onto the 96-well plate at a density of 5000 cells per well in 100 uL medium. The nanomachine solutions were lighted half an hour by the ultraviolet rays for sterilization and then diluted with fresh media to obtain different fractional concentrations—i.e., nondiluted (100 %) and diluted (1:7 [12.5 %], 1:3 [25 %], 1:1 [50 %]) extract solutions with Dulbecco’s Modified Eagle’s Medium (DMEM), respectively. Ten microliters of CCK-8 reagents were added into each well, and the plate was left standing for an additional 2 h at 37 °C. The optical density (OD) was measured using a multifunctional microplate reader (SpectraMax M5, Molecular Devices, Sunnyvale, CA, USA) at 450 nm wavelength. This process was repeated eight times in parallel. The results are expressed as the relative cell viability (%) with respect to a blank group only with a culture medium.

### Preparation of Urokinase Solutions

Urokinase solutions were prepared in the morning and stored at 4 °C until being used on the experiment day. Briefly, 250,000 units urokinase powder (Lizhu Pharmaceutical Factory, Guangzhou, China) was dissolved in 2 ml 0.9 % sterile saline, and the stock solution of 125 U/μl urokinase solution was made. Then, 1 ml of stock was diluted with 1 ml of saline to 62.5 U/μl urokinase solution, and 1 ml of 62.5 U/μl urokinase solution was diluted with 1 ml of saline to 31.25 U/μl urokinase solution. Using the same method, we obtained the final concentration of 15.625 U/μl solution. We then prepared final concentrations of 62.5, 31.25, and 15.625 U/μl urokinase solutions by making dilutions of the stock. Thus, 80 μl of the above dilutions was equivalent to 5000, 2500, and 1250 U of urokinase, respectively.

### Detection of d-Dimer

The d-dimer level of all samples was detected with an ACL TOP automatic coagulation analyzer (Beckman Coulter, Inc., AmericaBrea, CA, USA) using latex immunoturbidimetry. Each sample was assayed three times.

### Data Analysis

The data are presented as means ± standard deviation. All experimental data were the average of at least eight independent tests. All data were processed by SPSS (version 19.0; IBM, USA) software. The least significant difference (LSD) at the 5 % confidence level was used for comparing treatments.

## Results and Discussion

### The Hydrolysis Activity of the Reconstituted δ-Free F_o_F_1_-ATPase

When the chromatophore was incubated with LiCl, δ-subunit separated from b_2_-subunits of F_0_ and most of the F_1_ was removed from the c-ring rotor of F_0_. After the system was centrifuged three times, the remained protein and LiCl were removed. When the PS3 F_1_ purified from thermophilic bacteria *Bacillas* and the ε-subunit purified from *Escherichia coli* were added into the above resuspended system, the ATP synthase catalytic complex was assembled back to the F_0_ inset in the membrane of the chromatophore. The only difference was that the δ-subunit was not connected to the b_2_ and the rotation of F_1_ was no longer restricted by b_2_. As a result, the F_1_ motor rotated more easily.

**Fig. 2:**
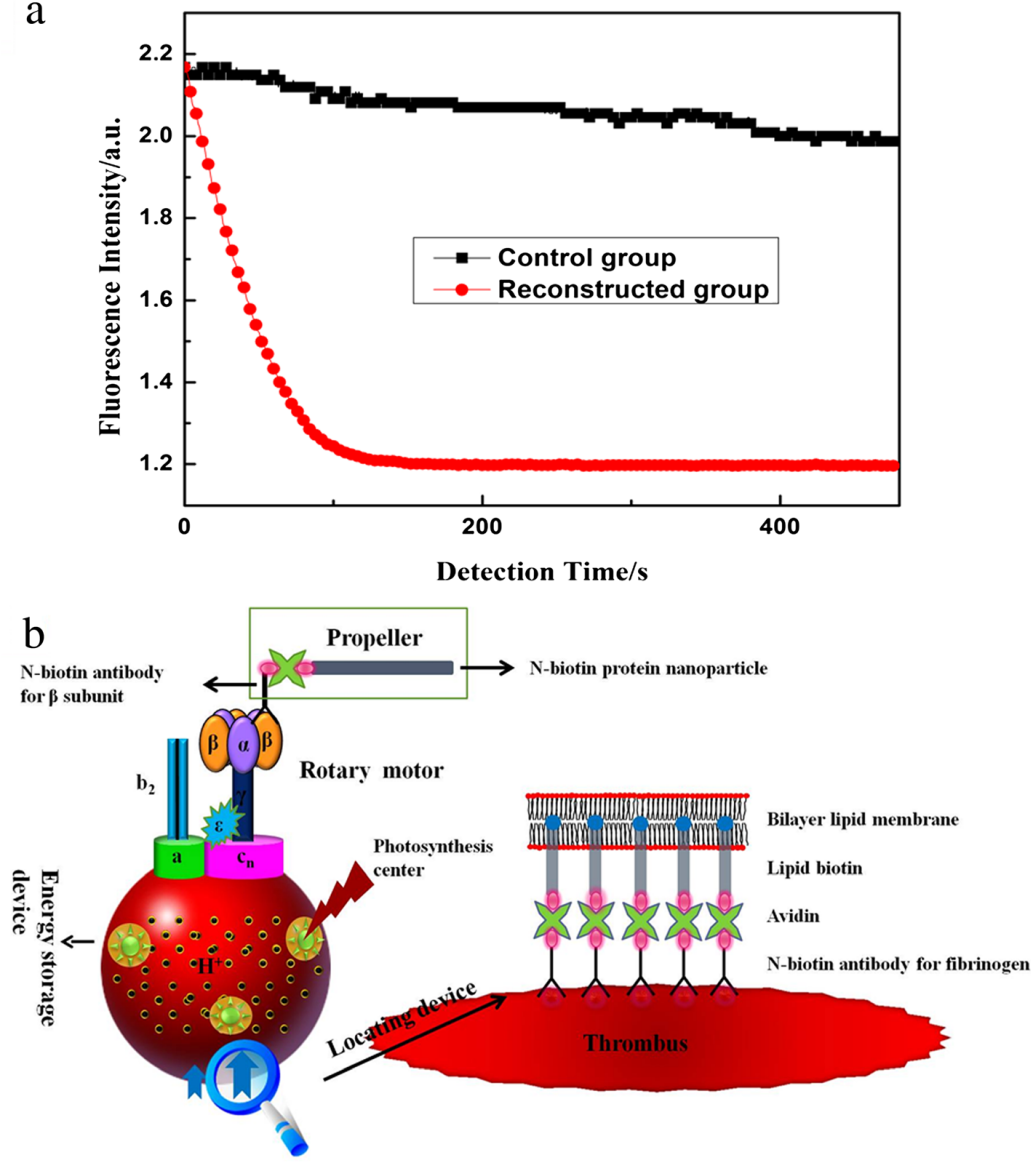
The hydrolysis activity of the native molecular motor and structure of the constructed nanomachine. **a** The hydrolysis activity of the native molecular motor compared to the reconstructed molecular motor. The *horizontal axis* shows time, and the *vertical axis* shows the fluorescence intensity, which is proportional to the concentration of NADH. The *square* matches the native F_0_F_1_-ATPase molecular motor, and the *circle* matches the reconstructed δ-free F_0_F_1_-ATPase molecular motor. **b** Structure of the constructed nanomachine. The nanomachine consists of four parts: rotary motor, energy storage device used for energy transformation and storage, locating device for combining fibrin present in the thrombi, and a propeller for accelerating thrombolysis. Light irradiation can stimulate the photosynthesis center to store energy in the nanomachine. The long propeller produced the stronger mixing power. The anti-fibrinogen antibody (locating device) can recognize and bind to fibrin within the thrombi

The curve in Fig. 2a indicates the disappearance of NADH and reflects the ATP hydrolysis rate of the two different systems. As shown in Fig. 2a, the reconstructed motor had better hydrolysis activity than the holoenzyme based on the usage of NADH and the ATP hydrolysis rate. This improvement in bioactivity provided a good foundation for the next step experiment.

### Installation of Propeller and Locating Device

This motor may generate stronger stirring power if a long chain is added to the β- or α-subunits. Therefore, we linked an anti-β-subunit biotinylated antibody to the β-subunit and then introduced avidin for binding to the biotin. Using this approach, a propeller with a greater length was bound to the molecular motor (Fig. 2b).

The vesicle wall of the chromatophore is composed of a lipid bilayer membrane. When lipid-biotin is mixed with the chromatophore, the lipid inserts into the membrane with the linked biotin extending outside the membrane (Fig. 2b). This method allows for binding of the anti-fibrinogen antibody containing avidin to the biotin on the chromatophore. This anti-fibrinogen antibody and the fibrinogen in blood clots can form a complex of antigen-antibody, which helps the nanomachine to bind to the thrombi and accelerates thrombolysis.

### Diameter and Zeta Potential

Table 1 shows the size distribution and zeta potential of the chromotophore and the nanomachine. The size of the nanomachine is about 110 nm (Additional file 1: Figure S1). It is a little bigger than chromatophore’s because of the avidin–biotin complex assembling on the surface layer of it. According to this study [24], the diameter of the avidin–biotin complex is about 5–10 nm, which is consistent with our results. As shown in Table 1 and Additional file 2: Figure S2, the zeta potential of the nanomachine measured in pH 7.4 water solution is about −23.4 mV, which was considered relatively stable [25].

**Table 1:** The size distribution and zeta potential of the nanomachine

Sample	Hydrodynamic diameter (*D*_h_) (nm)	Polydispersity index	Zeta potential (mV)
Chromatophore	99.89	0.192	−20.2
Nanomachine	110.2	0.251	−23.4

### Cell Viability of the Nanomachine

**Fig. 3:**
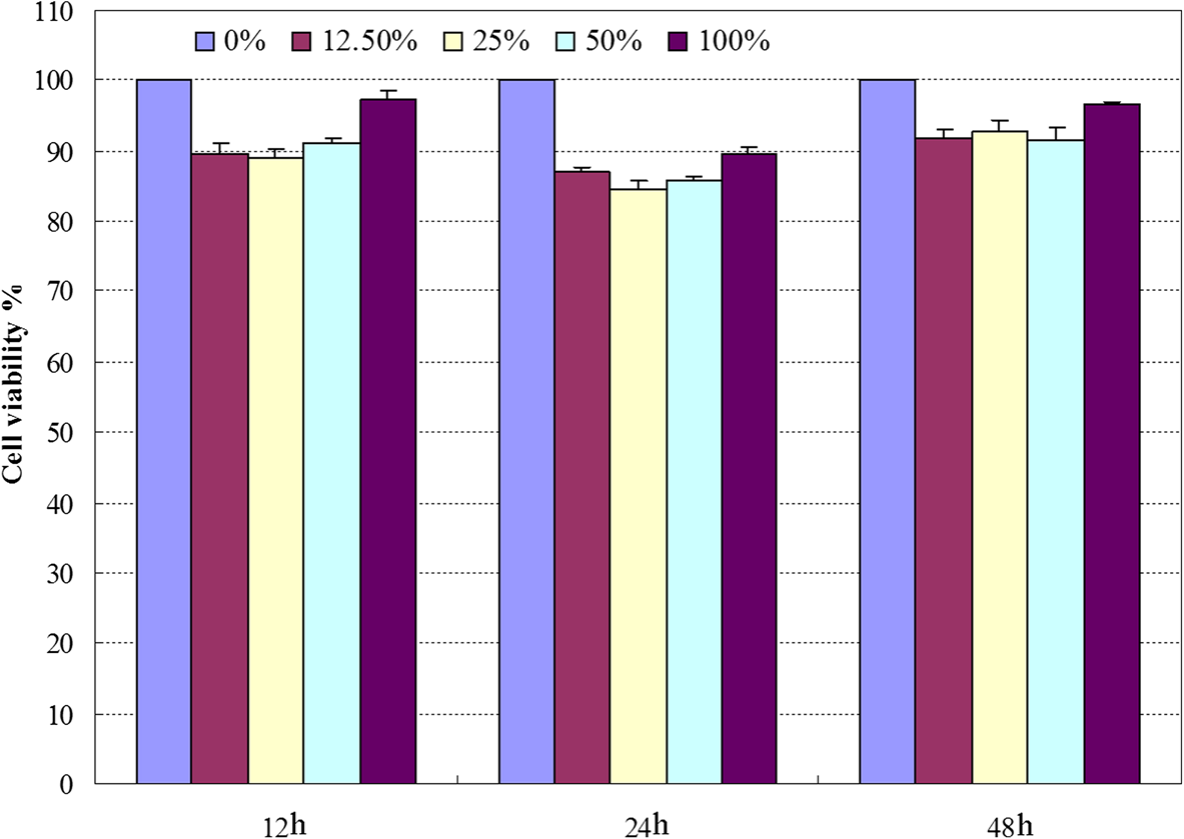
Cell viability of the nanomachine by CCK-8 assay with different concentrations of extract solutions

The cell viability of the nanomachine was evaluated using the CCK-8 assay which has a higher sensitivity and a better reproducibility than traditional MTT assay. As shown in Fig. 3, there is almost no cytotoxicity at various concentrations of the nanomachine and above 85 % of cells remained alive in 48 h. In addition, close to or more than 90 % of cells remained alive when incubated with the nondiluted (100 %) nanomachine solutions throughout the test, which means the nanomachine has no obvious cytotoxicity and has a good biocompatibility.

### Energy Storage in the Nanomachine

NaN_3_ and ATP were added to the nanomachine at a final density of 5 and 2 mM, respectively. Then, 40 μl of this nanomachine solution was irradiated with 600-nm light-emitting diode (LED) with a 560-nm filter for 40 min at 4 °C. NaN_3_ and ATP can fix the relative rotation of γ and α_3_β_3_ and form a new and combined rotor α_3_β_3_γεc_n_ which rotates more freely as a whole. The molecular motor began to rotate in one direction as protons transported from the outside of the chromatophores into the inner region and then a proton motive force was established. The motor began to rotate in the reverse direction when the irradiation was removed and the temperature reached room temperature. The contrasting movements resulting from energy storage and release accelerated thrombolysis efficiently.

### In Vitro Acceleration of the Nanomachine in Urokinase-Mediated Thrombolysis

**Fig. 4:**
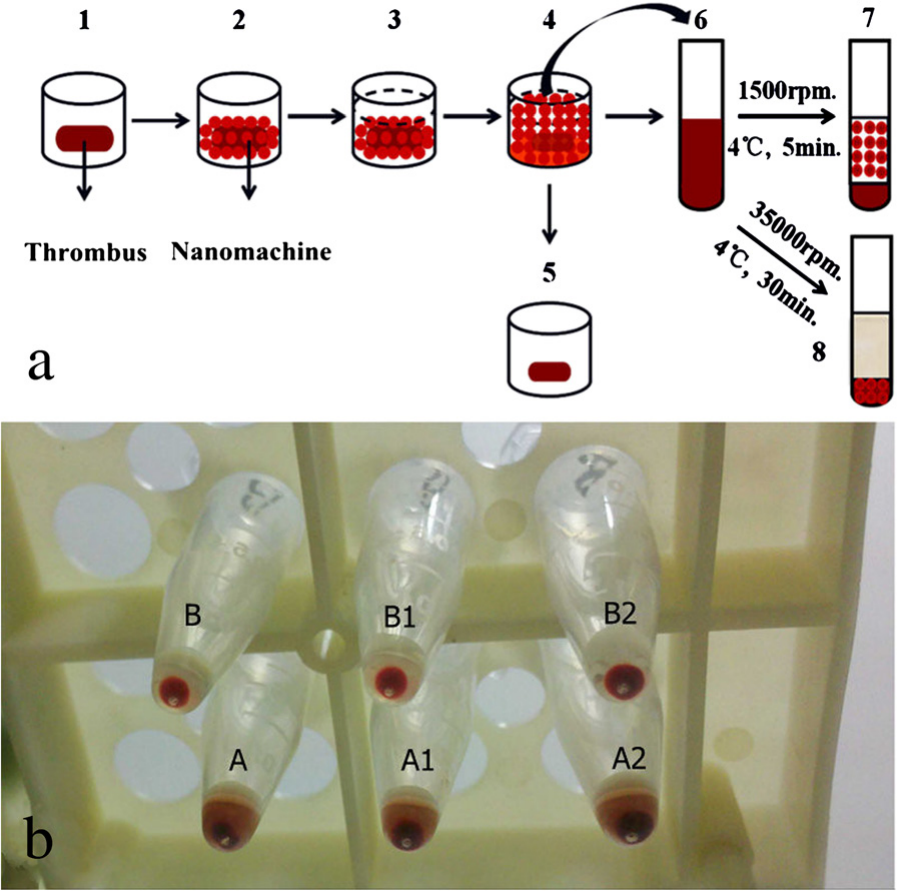
Flow chart of thrombolysis process and relative level of thrombolysis. **a** A flow chart of thrombolysis process. *1* A blood clot was placed in a well of a 96-well plate; *2* the nanomachine was added directly onto the blood clot and the locating device reacted with the blood clot; *3* urokinase (80 μl) was added into the well; *4* thrombolysis; *5* residual blood clot; *6* liquid mixture after thrombolysis; *7* liquid supernatant after centrifugation for 5 min at 1500 rpm; and *8* liquid supernatant without the nanomachine after centrifugation for 30 min at 35,000 rpm. **b** The relative level of thrombolysis. Group A with nanomachine and group B without nanomachine (control group). The liquid mixture in chart was centrifuged at 1500 rpm

Figure 4a provides a schematic diagram of the experimental process. The bar thrombus was cut into equal sections and washed with normal saline three times. The sections were then placed in individual wells of a 96-well plate, and 10 μl nanomachine solution was carefully placed on the surface of each thrombus ensuring full contact and isopyknic 0.9 % sterile saline was added in control groups. Then, 80 μl urokinase was added into the well and incubated for 1.5 h at room temperature. Each thrombus was found resolved slowly during the incubation time. At the end of the incubation, all of the liquid, except the remaining block, was removed and injected into Eppendorf (EP) tubes. The composition of the liquid contains degradation products of fibrin and red blood cell fragments.

We then centrifuged all tubes at 1500 rpm and 4 °C for 5 min. Through this approach, the fragments of thrombolysis that had been dissolved sank to the bottom of the tube, while the nanomachine remained suspended in the solution. The results were shown in Fig. 4b. Series A samples were nanomachine groups while the series B were control groups. For A1, A2, and A3, the concentration of urokinase was 1250, 2500, and 5000 U, respectively. Series B had the same concentrations orderly. Series A samples had three layers and the nanomachines settled in the middle. Series B had only two layers: supernatant and precipitate. The volume of precipitate can reflect the extent the blood clots were dissolved, which suggests that the larger volume of the bottom sediments is, the more thrombolysis of the clots have. Based on visual inspection, we found that A>B, A1>B1, and A2>B2, which demonstrates that the nanomachine facilitates thrombolysis of clots by effectively stirring in the urokinase solution of different concentrations.

**Fig. 5:**
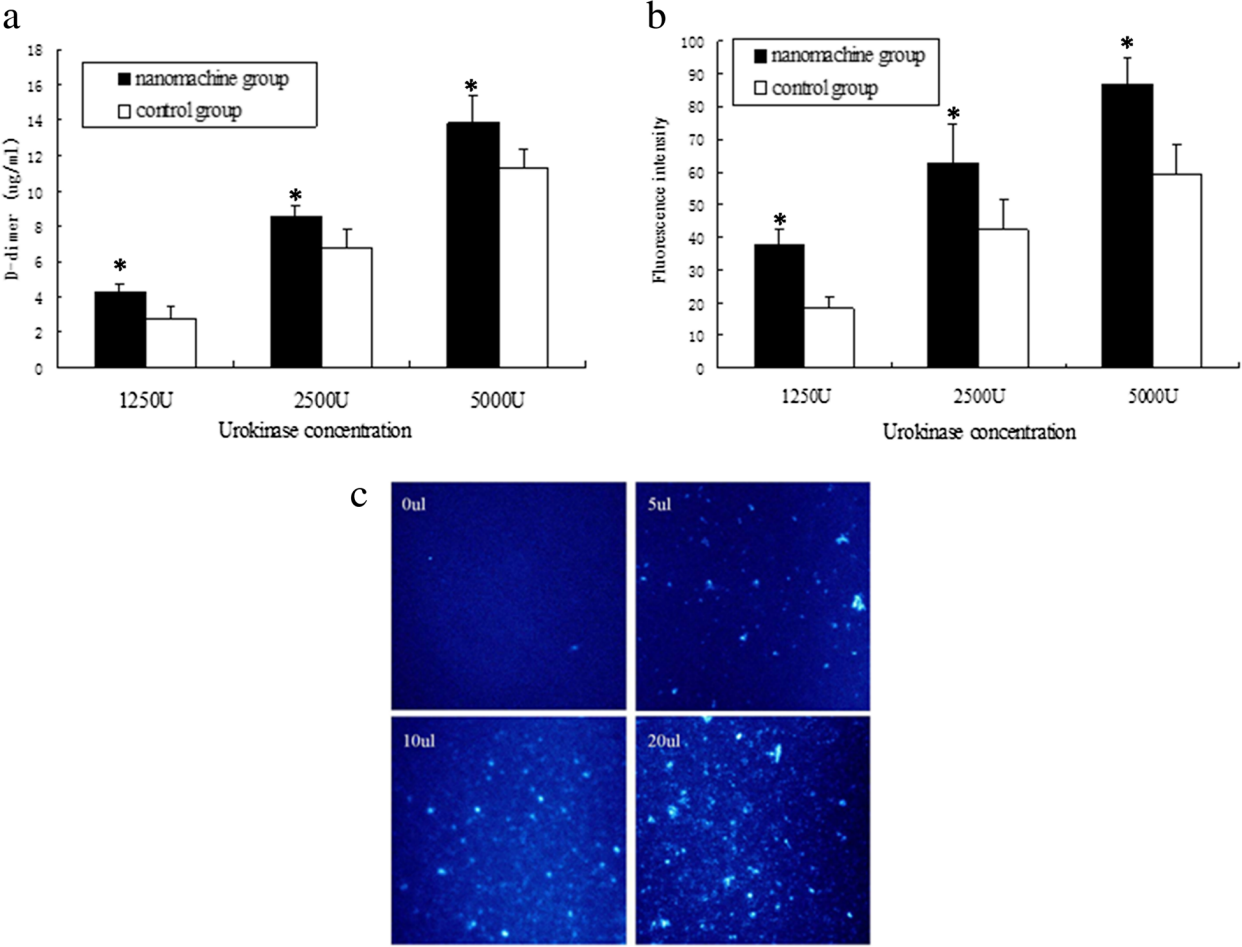
The concentration of d-dimer, fluorescence intensity of thrombolysis, and fluorescence microscope images of the supernatant. **a** The concentration of d-dimer in the supernatant of each subgroup. Data are shown as mean + standard deviation. All A series of samples are nanomachine groups and B series are control groups. *P <0.05 compared with the control groups. **b** The fluorescence intensity of thrombolysis in the supernatant after the system were centrifuged for 30 min at 35,000 rpm. Data are shown as mean + standard deviation. The A series contains the nanomachine and B series contains the control groups. *P <0.05 compared with the control groups. **c** Fluorescence microscope images of the supernatant after thrombolysis assisted by 0, 5, 10, and 20 μl nanomachines

We performed further quantification to confirm the above results, whereby 25 μl supernatant aspirated from the above EP tubes were diluted with the same volume physiological saline and then centrifuged at 3000 rpm for 15 min. The d-dimer in the supernatant was then assessed by the automatic coagulation analyzer. Table 2 shows the group assignment and detailed results of the d-dimer. The test was repeated eight times and presented as the average values of each subgroup (Fig. 5a). The d-dimer is the specific biomarker in terminal degradation products of cross-linked fibrin, which is the main part of thrombus and can be dissolved by urokinase. The d-dimer level increases as the cross-linked fibrin degradation of the clot increases, and the changes of plasma d-dimer content has already been recognized as the symbol of thrombolysis [26]. Lawler et al. found that if the thrombolysis effect was reached, the d-dimer level increased rapidly. Thus, plasma d-dimer level can be used as an important observation index in treatment effectiveness of thrombolysis [27]. Therefore, we can quantitate the degree of thrombolysis by measuring the d-dimer level in the solution. As shown in Fig. 5a, the levels of d-dimer in all of the nanomachine subgroups were higher than the urokinase alone groups, illustrating that the nanomachine accelerates the lysis of clots at different urokinase concentrations.

**Table 2:** The concentration of d-dimer in different subgroups (mg/L)

Concentration of urokinase (U)	Nanomachine group	Control group
1250	4.25 ± 0.53*	2.83 ± 0.67
2500	8.61 ± 0.57*	6.80 ± 1.02
5000	13.89 ± 1.43*	11.3 ± 1.06

The levels of d-dimer in all of the nanomachine subgroups were higher than the control groups

*P <0.05 compared with the control group

In order to confirm these results, we tested the nanomachine effectiveness with FITC-labeled thrombus using another semi-quantitative method. Ectogenic fibrinogen labeled by FITC was mixed with whole blood obtained from healthy volunteers and the thrombus with fluorescence was made as described above. The experimental procedure followed that outlined in Fig. 4a until step 6, at which point the dissolved solution was placed at 4 °C and centrifuged at 35,000 rpm for 30 min. During centrifugation, the fragments from thrombolysis and the nanomachines precipitated, and the material labeled by FITC stayed in the supernatant. Therefore, the FITC-labeled fibrin, which had been uniformly dispersed in the buffer after nanomachine-mediated thrombolysis, was measured by separating the supernatant from the red blood cells and nanomachines through centrifugation. It is possible to assess the effects of increasing thrombolysis by the nanomachine by comparing the fluorescence intensities between groups. The stronger the fluorescence intensity is, the more obvious the effect of thrombolysis will be. Figure 5b shows the comparison of fluorescence intensities between the nanomachine groups and the control groups, whereby 10 μl suction from the supernatant was spotted and assayed by a fluorescence detector using a 480-nm-wavelength diode as the excitation source. The mean values are shown in Fig. 5b and Table 3. We found that the fluorescence intensities of supernatants increased as the urokinase concentration increased and that the nanomachine groups had higher fluorescence than the control groups (A2>B2, A1>B1, and A>B, respectively). Taken together, these findings confirmed that the nanomachine accelerated the thrombolysis at different concentrations of urokinase.

**Table 3:** The values of fluorescence intensities in different subgroups

Concentration of urokinase (U)	Nanomachine group	Control group
1250	37.6 ± 4.6*	18.3 ± 3.8
2500	63.0 ± 11.7*	42.3 ± 9.0
5000	87.0 ± 7.8*	59.7 ± 8.5

The nanomachine groups had higher fluorescence intensities than the control groups

*P <0.05 compared with the control group

Figure 5c shows the results of thrombolysis of urokinase incubated with increasing amounts of nanomachines by fluorescence microscopy. To determine whether the thrombolytic effect was strengthened by the nanomachines, we added an increasing amount of nanomachines (0, 5, 10, or 20 μl) to the system of 1250 U urokinase and the same volume of FITC-labeled thrombus. After the thrombolysis, 35,000 rpm centrifugation was conducted and 5 μl of supernatant was spotted on an aseptic glass plate. As shown in Fig. 5c, white spots represent lytic thrombus fragments. The brightness, number, and diameter of these spots were compared visually under a high-power fluorescence microscopy. We found that the nanomachines increased the rate of thrombolysis of urokinase in a concentration-dependent manner, as indicated by a greater number of blood block fragments and fluorescence intensity.

In the previous studies about assisted thrombolysis, the researchers had used a variety of methods to try to carry the encapsuled drugs to reach the thrombus site, such as liposomes and magnetic nanoparticles (NPs). But as described in some reports, the use of liposomes was limited by poor repeatability and low encapsulation efficiency and enzyme inactivity, while magnetic NPs was limited by sizes and complicated controllability [28–30]. In this work, we used F_0_F_1_-ATPase as a mechanical rotation motor. We separated all the subunits from it first, then reassembled and optimized, and obtained a new relatively complicated nanomachine at last. Under the condition of light driving, the new nanomachine can produce proton gradient, which provides energy for the nanomachine, and makes it rotate smoothly and easily without the limitation of the δ-subunit. Although the assembled process of the δ-subunit-free F_0_F_1_-ATPase is relatively complicated, it has good repeatability, stability, and activity according to our previous studies [14, 15, 22]. However, the proton gradient formed by the light driving may be not the only selection. Li et al. entrapped glucose oxidases (GODs) into the cavity of polymeric hollow spheres of biomimetic nanocapsules, which can hydrolyze glucose in the suspension and produce proton gradient, thus supply power for ATPase. In comparison to our light-driven proton pump, this type of biomimetic capsule for ATP synthesis maybe a cheaper and better choice for the future study [31].

The application of nanotechnology to assisted thrombolysis has been a research hotspot in the field of thrombolysis. In this experiment, the reassembled nanomachine showed preferable assisted thrombolysis effect. It is known that δ-subunit plays a switch role between the F_0_ and F_1_ in the ATPase and restricts the efficient rotation of F_1_. The δ-subunit-free F_0_F_1_-ATPase could create proton potential energy under 4 °C continuous irradiation. The proton gradient is an important basis to ensure F_1_ to rotate as the rotor efficiently. The propeller device amplifies the mechanical force like an extended mechanical arm and results in a stirring effect in the process of thrombolysis, which can make full contact between urokinase and thrombosis, and increase urokinase solution infiltrating into thrombus, thus accelerates the process of thrombolysis.

Many problems such as biological toxicity and immune reaction should be taken into account in the application of nanotechnology in medicine. In this study, the materials and buffer used to assemble nanomachine were simulated in physiological environment of human body. The cell viability evaluated using CCK-8 assay shows that the nanomachine almost has no cytotoxicity at various concentrations, assuring its good biocompatibility. Thus, we could have a try to use this nanomachine in animal thrombolytic test in the future. In animal experiments, the researchers tried to improve the assembly technique, develop intelligent drug delivery system, fabricate nanotechnology-based products, make the nanoparticles right sizes, and want to get the biodegradable materials to encapsulate the drugs; all these works were efforts to reduce adverse reactions [32–35]. In the future studies, in order to avoid the above complicated restrictions and unpredictable results, we plan to use electrodeposition coating technology [36] to fix the assembled nanomachine to the tip of arterial catheter during the interventional operation to help recanalization in carotid artery occlusion model in New Zealand rabbits. Even for animal experiment the nanomachine could not be retained in vivo, thus, we could avoid the biological toxicity and immune reaction and other adverse effects which could not be detected at present as far as possible.

Although there are many exciting developments in the fields of nanotechnology and nanomedicine, they are still in the early stages. It is envisaged that developments in nanotechnology will have an enormous impact on the field of thrombotic diseases. Much of the ongoing work should be done in the future studies.

## Conclusions

A δ-subunit-free F_0_F_1_-ATPase motor was successfully created by reconstructing the original chromatophore extracted from *R. rubrum*. Energy was stored when the motor was exposed to 600-nm-wavelength irradiation at 4 °C and released when irradiation removed and followed by a slow rise to room temperature. Both the energy storage and release process promoted the rotation of the nanomachine. The new combined propeller and the thrombus-locating device in the motor formed a more complicated nanomachine which has a good stability and no cytotoxicity. This new nanomachine had better thrombolysis effect than free urokinase.

Though additional studies are required to determine the best way to optimize the reconstruction to get a good biocompatible nanomachine which can be observed in vivo, the new nanomachine which using less thrombolytic drugs but getting more efficient thrombolysis brings us a promising research idea in the field of thrombolysis study.

## Additional files

Additional file 1:
**Size distribution by intensity.**
Additional file 2:
**Zeta potential distribution.**
