# Supplementary figures and images for: Visible Thrombolysis Acceleration of a Nanomachine Powered by Light-Driving F0F1-ATPase Motor
Source: Nanoscale Res Lett. 2015 May 21;10:227. doi: 10.1186/s11671-015-0918-z (PMC4447733; doi:10.1186/s11671-015-0918-z)

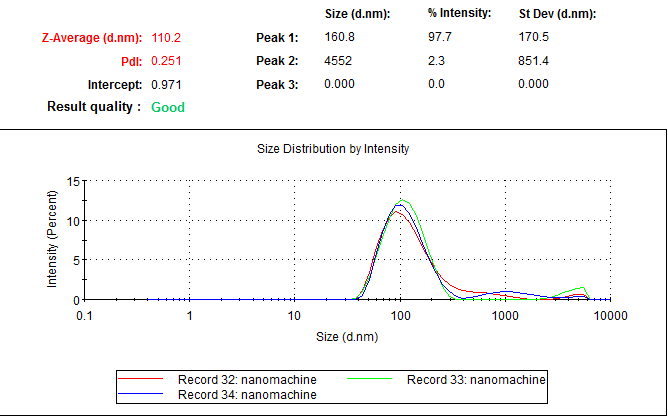

Supplement: Additional file 1: — Size distribution by intensity. [file 11671_2015_918_MOESM1_ESM.zip › 11671_2015_918_add1.bmp]

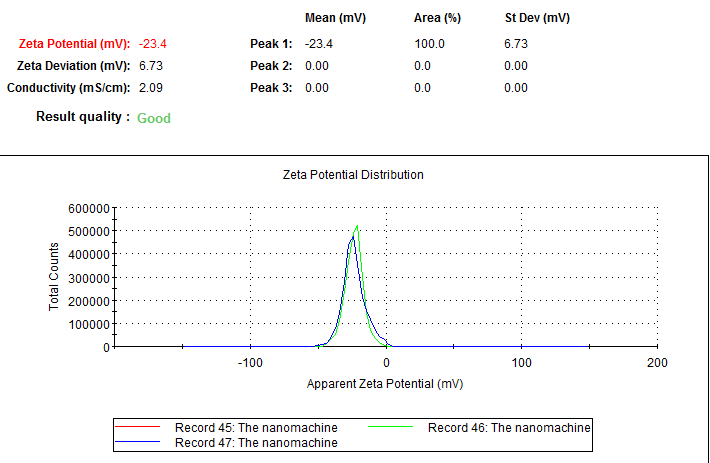

Supplement: Additional file 2: — Zeta potential distribution. [file 11671_2015_918_MOESM2_ESM.zip › 11671_2015_918_add2.bmp]
